# Supplementary material for: Grp94 Inhibitor HCP1 Inhibits Human Dermal Fibroblast Senescence
Source: Genes (Basel). 2022 Sep 14;13(9):1651. doi: 10.3390/genes13091651 (PMC9498348; doi:10.3390/genes13091651)
Supplement: Supplementary file 1 [file genes-13-01651-s001.zip › genes-1888071-supplementary/genes-1888071-supplementary.pdf]

Supplementary methods

Cell viability assay

HDFs were seeded onto 96-well plates, and then treated with 0.1% DMSO (as control) or HCP1 at indicated concentration for 48 h. Cell viability was determined by sulforhodamine B (SRB) assay according to the manufacturer's instructions.

Supplementary figure and figure legend

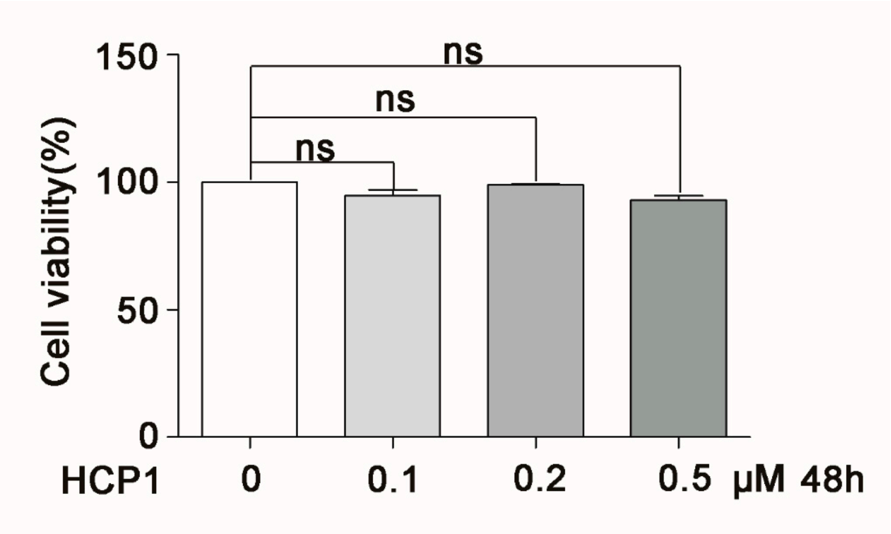

Figure S1. The senescent HDFs were treated with different concentrations of HCP1 for 48h, and the cell viability was detected by SRB.
